# Carbon/Silicone Nanocomposite-Enabled Soft Pressure Sensors with a Liquid-Filled Cell Structure Design for Low Pressure Measurement

**DOI:** 10.3390/s21144732

**Published:** 2021-07-10

**Authors:** Fei Wang, Xiaoming Tao

**Affiliations:** Research Institute of Intelligent Wearable Systems, Institute of Textiles and Clothing, The Hong Kong Polytechnic University, Hung Hom, Hong Kong 999077, China; 11902640R@connect.polyu.hk

**Keywords:** soft pressure sensors, structural design, hysteresis, conductive nanocomposite, artificial skin

## Abstract

In the fields of humanoid robots, soft robotics, and wearable electronics, the development of artificial skins entails pressure sensors that are low in modulus, high in sensitivity, and minimal in hysteresis. However, few sensors in the literature can meet all the three requirements, especially in the low pressure range (<10 kPa). This article presents a design for such pressure sensors. The bioinspired liquid-filled cell-type structural design endows the sensor with appropriate softness (Young’s modulus < 230 kPa) and high sensitivity (highest at 0.7 kPa^−1^) to compression forces below 0.65 N (6.8 kPa). The low-end detection limit is ~0.0012 N (13 Pa), only triple the mass of a bee. Minimal resistance hysteresis of the pressure sensor is 7.7%. The low hysteresis is attributed to the study on the carbon/silicone nanocomposite, which reveals the effect of heat treatment on its mechanical and electromechanical hysteresis. Pressure measurement range and sensitivity of the sensor can be tuned by changing the structure and strain gauge parameters. This concept of sensor design, when combined with microfluidics technology, is expected to enable soft, stretchable, and highly precise touch-sensitive artificial skins.

## 1. Introduction

Recent years have seen extensive research in humanoid robots [1], soft robotics [2,3], prosthetic limbs [4], as well as wearable electronics [5]. All these fields are in great need of reliable soft pressure sensors which, when in the form of a sensor matrix or as an artificial skin, may enable many devices with new functionalities. For example, touch-sensitive humanoid robots with improved safety for human–machine interaction [1], soft robotics with real-time feedback of force and position [6], prosthetic limbs with tactile perceptions [4], as well as pressure-sensitive mannequins for garment comfort [7] and compression evaluation [8]. Therefore, a growing body of research has been conducted on soft pressure sensors [9]. The flexible capacitive pressure sensor with micro-structured rubber dielectric layers [10] measured a pressure as low as 3 Pa. The transparent triboelectric and self-powered pressure sensor [11] was sensitive to 0.4 Pa with a low-end detection limit of ~13 mPa. Further, the flexible pressure-sensitive organic thin film transistors [12] exhibited a maximum sensitivity of 8.4 kPa^−1^ in the low pressure range. On the other hand, soft pressure sensors with superior deformation capabilities have also been developed. A soft pressure sensor using embedded micro-channels and liquid conductors [13] was functional up to strains of ~250%, and pressure sensors with carbon-nanotube-inlaid conductors [14] were functional up to strains of 300%, which are well beyond the 44.6% strain limit of the human skin [15]. These highly stretchable sensors, however, were insufficient in pressure sensitivity. In 2020, the liquid metal conductor-based pressure sensor [16] was made thinner, but its sensitivity was not improved much.

An ideal artificial skin should be not only be touch-sensitive, but also as soft as the human skin, so as to ensure accuracy, safety, and comfort in human–machine interaction. Indeed, to emulate the pressure sensation of a natural skin, large arrays of soft pressure sensors on a flexible and stretchable substrate are required [10]. Therefore, it is vital to include both high sensitivity and low modulus. Unfortunately, few sensors in previous research satisfied both. Most researchers put too much emphasis on high or even ultrahigh sensitivity, despite that excessively high sensitivity can introduce more noise and make calibration difficult, leading to lower measurement accuracy.

Apart from high sensitivity and low modulus, low hysteresis is also crucial for precise pressure measurement. In soft resistive sensors, hysteresis and resistance relaxation are interrelated, both of which arise from the viscoelastic ingredients of materials. In fact, resistance relaxation or resistance viscosity [17] remains one of the greatest challenges that hamper soft resistive sensors from providing accurate measurements [18]. Fan et al. reported liquid-capsule flexible pressure sensors with a hysteresis of 11.5% [19], which, however, are only partly soft. In 2019, Liang et al. further demonstrated an elastomeric pressure sensor using Cr/Au strain elements with negligible hysteresis. Unfortunately, the sensor was made from polymer with a modulus of 2 MPa [6], two orders of magnitude larger than that of the human skin (30–70 kPa) [20]. In 2020 and 2021, wearable capacitive pressure sensors that are flexible [21] or highly elastic [22] both demonstrated a hysteresis virtually close to zero. The key sensing materials were ionic gel/Au-coated pillars and carbon black/elastomer, respectively. However, these capacitive sensors are relatively demanding in electromagnetic shielding and complex in readout circuits.

Therefore, few of the previous sensors are approaching the force receptors of the human skin in terms of both sensitivity and softness. It remains challenging to acquire high sensitivity, softness, and low hysteresis altogether for one pressure sensor, especially in the low pressure range (<10 kPa). This article will try to address this issue by studying a cell-structured soft pressure sensor. A benchmark of our bio-inspired sensor design against typical sensors in the literature is given in Table 1.

## 2. Concept of Sensor Design

### 2.1. Design Strategy

One of the effective design strategies for pressure sensors is to convert pressure into strain by using a conversion structure and then measure signals from the strain gauge. The strategy endows the traditional rigid load cells with high accuracy and low cost [28], by virtue of the well-established strain gauge technology. The strategy, combined with fabric strain gauge technology, has also been applied to the design of soft pressure sensors. In 2011, soft pressure sensors with a conversion structure of two tooth-structured silicone layers and a fabric strain gauge in them were demonstrated sensitive to a pressure between 0 and 2 MPa [29]. Furthermore, in 2014, a conversion structure of silicone pillar enabled soft pressure sensors for high-speed impact force monitoring. The sensitivity was 1 MPa^−1^ for a pressure up to 8 MPa [30]. Unfortunately, it is difficult for the previous conversion structures to convert low pressure below 10 kPa into measurable strain, due to the limitation of material hardness and strain gauge. Therefore, it is necessary to design a new conversion structure.

### 2.2. Inspiration

**Figure 1:**
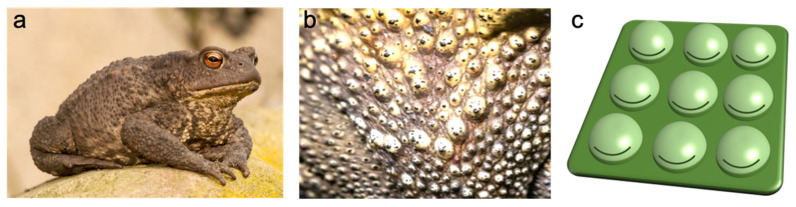
(**a**) Picture of a toad. (**b**) Warty texture of a toad’s skin. (**c**) Schematic of bioinspired pressure sensor arrays with spherical bumps. The black lines are strain gauges.

The conversion structure of our sensor is inspired by the skin of toads. Although both frogs and toads are amphibians, frogs have thin and smooth skins, whereas the skin of a toad (Figure 1a) is always dry and warty-looking (Figure 1b). Under the warty bumps, there are mucous and granular glands, which are responsible for the production of mucus and toxic secretions, respectively [31]. The mucous glands are smaller and their secretory portion is composed of a cuboidal or columnar epithelium, while the larger granular glands are made up of a gland alveolus formed by a secretory layer covered externally by a myoepithelial layer. Such warty-looking morphology inspired us to explore artificial skin dotted with spherical bumps (warts), which are actually pressure sensors protruding out from the surface. Figure 1c is a schematic drawing of the pressure sensor arrays. There is a resistive strain gauge on each sensor unit. Because the bioinspired design is no longer confined to a flat surface, it shall facilitate small pressure measurement and possibly three-dimensional pressure mapping—challenging tasks for conventional pressure sensors.

### 2.3. Analysis of Conversion Structure

The spherical bumps, i.e., the conversion structures of the pressure sensor, include at least three types of design: hollow (air-filled), cell (liquid-filled), and solid structures. In this research, a liquid-filled cell structure was chosen because it will endow the pressure sensor with large deformation and high sensitivity. (1) The deformation of the outer surface can be larger than that of hollow structures, because oil is incompressible and air is compressible. (2) It will be much more sensitive to small pressures than a solid structure. Owing to the zero shear modulus of liquid, the nominal elastic modulus of the structure can be much lower, and the structural deformation is primarily membrane tensile deformation rather than compression of a monolithic solid block. (3) According to Pascal’s law, pressure change at any point in a confined fluid at rest is transmitted undiminished to all points in the fluid, which theoretically ensures a uniform latitudinal deformation and an effective transfer function from pressure to strain. (4) When liquid volume exceeds that of a hemisphere, the conversion structure will have an equator which, in theory, possesses a larger latitudinal strain under compression, hence a higher sensitivity.

## 3. Hysteresis of the Materials for Strain Gauge

Many materials for soft strain gauges have been developed, including Ag nanowire aerogels [32], stick-on hydrogel [33], cellulose nanocrystalline hydrogel [34], carbon black/Ecoflex^TM^ (Smooth-On, Inc., Macungie, PA, USA) composite [35], carbon nanotube/styrene-butadiene-styrene [36], as well as carbon black/silicone composite [19]. Herein, a carbon/silicone nanocomposite developed in our previous research [37] will be printed onto the conversion structure as strain gauges, owing to its sufficient sensitivity, good linearity, and excellent repeatability for a strain up to 50%, as well as its low cost and compatibility with silicone. Despite that, while it may increase hysteresis of the pressure sensors, hysteresis of this nanocomposite stain gauge has not been fully understood yet,. Therefore, we first explored the electromechanical hysteresis of the carbon/silicone nanocomposite in a material’s perspective.

### 3.1. Preparation of Composite Samples

Materials of the nanocomposite include carbon black (CB, Carbon^®^ ECP600JD, Akzo Nobel, Amsterdam, The Netherlands), silicone elastomer (SE, ELASTOSIL^®^ LR6200 A and B, Wacker Chemie AG, Munich, Germany), and silicone oil (SO, nontoxic dimethyl, Che Scientific Co., Kwai Chung, Hong Kong, China).

In total, 25 samples were made from SE, SE/SO, and SE/SO/CB, as listed in Table 2. The control groups of SE and SE/SO were included to help ascertain the origin of hysteresis. Uniform mixtures of each formulation were cast onto a polytetrafluoroethylene (PTFE, Teflon^®^, Chemours, Wilmington, DE, USA) mold, flattened with a draw knife, and vulcanized in a convection oven at 100 °C for 25 min (SE) or 15 min (the other formulations). Samples #11–#15 and #21–#25 were further heated at 100 °C for 34 h to ensure thorough evaporation of SO, as, after 34 h, the mass remained unchanged. Because all the samples were made using identical molds, they were of the same initial dimension, 0.5 mm × 50 mm × 20 mm. When SO evaporated, the composite shrank in dimension and decreased in weight accordingly. The shrinkage of samples #11–#15 was slightly higher than that of samples #21–#25, due to the existence of porous CB nanoparticles that hold the SO in the latter group.

**Figure 2:**
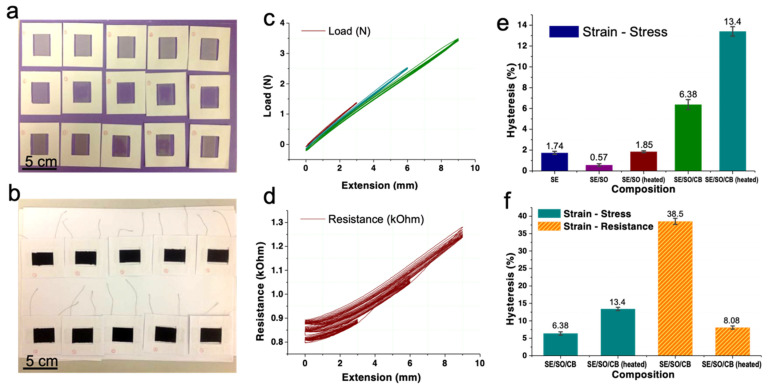
(**a**,**b**) Pictures of sample #1 to #25 fixed in cardboards. They were rowed as in Table 2. (**c**) The extension-load curves of sample #1. (**d**) the extension-resistance curves of sample #25. (**e**) Mechanical hysteresis of all the samples. (**f**) Comparison between mechanical and electromechanical hysteresis of the samples.

After mold release, the samples were fixed between two cardboards with 30 mm × 25 mm hollow areas. For each conductive sample, two silver plated nylon yarns (70D × 2) were parallel glued in advance on one cardboard at a distance of 36 mm. Figure 2a,b shows all the 25 samples held in cardboards.

### 3.2. Testing Method

The samples were tested using a coupled electromechanical setup, including an Instron 5944 (Instron Corp., Binghamton, NY, USA), an integrated versa channel, a voltage divider (excitation voltage was 10 V), and a computer for data acquisition, display, and storage. The tensile deformation and force, and the output voltage of the samples, were measured by Instron and the versa channel, respectively, in a synchronized manner. Electrical resistance of the sensor can be derived according to formula *R_sen_* = *R_ref_* × *V*/(10 − *V*), where *V* is the output voltage of the sensor. The reference resistor was 1 MΩ.

The samples were mounted between two clamping heads on Instron 5944. Once on Instron, the two arms of cardboards were cut off. The sample gauge length was 30 mm. All the samples were given 3 mm (10% strain), 6 mm (20% strain), and 9 mm (30% strain) extensions for 10 cycles, respectively. The extension speed was 60 mm/min and there was an interval of 6 s when the maximum extension distance changed. Extension-load and extension-resistance curves for the 25 samples were plotted. Typical curves are displayed in Figure 2c,d. Hysteresis will be calculated according to the 2nd cycle at each tensile strain because, from the 2nd cycle on, the hysteresis became stable.

### 3.3. Mechanical Hysteresis

Figure 2e depicts the mechanical hysteresis of all the samples. Hysteresis of pure SE was 1.74% due to its weak plasticity. When SO was added, it dropped to 0.57% because the smaller SO molecules saturated in the cross-linked PDMS network serve as a lubricant, weakening the internal friction. After heat treatment, hysteresis rebounded to 1.85% due to the volatilization of SO and increased porosity of the nanocomposite.

In comparison, hysteresis of CB/SE/SO was large at 6.38%, indicating the complex mechanical interactions between PDMS network and CB aggregates with fractal structures. The SO inside CB/SE/SO may have alleviated this friction or interaction. After further heat treatment, a lot of the SO volatilized. Consequently, the hysteresis of CB/SE/SO surged to 13.4%, which implies higher energy dissipation. For the CB/SE/SO samples, this dissipation was believed to be related with the slippage of entanglements in the transition layer between the CB-bounded SE layer and the mobile SE layer phase [38], and partially the release of elastically immobilized SE trapped within the CB filler network or agglomerates [39].

### 3.4. Electromechanical Hysteresis

Figure 2f compares the mechanical and electromechanical hysteresis of CB/SE/SO samples with and without a thorough heat treatment. Before analyzing the results, we need to consider the conduction mechanism and the morphology of the CB/SE/SO nanocomposite. (1) The conductive fillers, i.e., the carbon blacks, formed an electrically conductive network according to the percolation theory [40]. The resistance was determined by the shortest effective conductive path. There was destruction and formation of the paths (breakdown and re-agglomeration of filler–filler bonds) at every moment during the cyclic tensile tests, which made the conductive paths random and complex. The ever-changing conductive paths were directly related to real-time strains. (2) The electrical conduction between adjacent CB aggregates or agglomerates was dominated by the electron tunneling effect [41] as electrons could go through a potential barrier in a thin insulating film [42]. In tunneling resistance, the neighboring conductive particles needed to be close enough; its cut-off distance was called electron-tunneling distance [43]. The distance was generally below 10.0 Å [43,44]. (3) The SO molecule used here had a volume of 225.64 Å^3^, while the CB particle was approximately 30 nm in diameter (1.1 × 10^8^ Å^3^ in volume) with a void volume over 80%. Therefore, it was easy for the SO or part of the SE molecules to penetrate into the CB particles before vulcanization, forming a stable interface between the CB and the matrix after vulcanization. The penetration of SO and SE into CB was proven through scanning electron microscope in our previous study [37].

The SO molecules were small enough to fit both inside and between CB aggregates, but the nanocomposites could remain conductive since the dimension of the SO molecule is below the cut-off distance of 10.0 Å. The SO molecules between CB helped to reduce the friction. So, the mechanical hysteresis increased from 6.38% to 13.4% after baking. The 38.5% hysteresis in resistance was sixfold that of the mechanical hysteresis, which should be a result of the flow of SO. The SO molecules between neighboring CB particles or aggregates may provide electron tunneling resistance at 0% strain. However, after a tensile cycle, the SO inside the CB or from elsewhere may come into the interface, disabling the tunneling. Alternatively, the shape and structure of CB aggregates changed due to flow of SO. Thus, there was destruction and formation of the paths in a complicated manner. Interestingly, hysteresis dropped significantly to only 8.08% after heat treatment, which was even smaller than the 13.4% mechanical hysteresis of the same material. The reason could be that, after volatilization of the SO, CB particles and aggregates were easy to come into direct contact with each other during tensile tests, making the reformation of conductive paths quicker, hence ensuring less viscosity in resistance.

Therefore, heat treatment helped to decrease the electromechanical hysteresis by removing excess SO. The lowest hysteresis was 8.08%. As a consequence, the hysteresis limit of the cell-structured pressure sensors should be around 8.08%.

## 4. Materials and Fabrication of Pressure Sensors

### 4.1. Materials

Polydimethylsiloxane (PDMS, SILASTIC^®^ 3483 Mold Making Rubber, Dow Corning, Midland, MI, USA), was used to construct the conversion structure. This PDMS has an elongation at break as high as 600% and its Young’s modulus was 230 kPa, approaching that of the human skin. The CB/SE/SO nanocomposite was used as strain gauges, owing to its advantages as explained in Section 3. Silver-coated nylon yarns (SCNY) (70D × 2/24F/2, Xiamen Unibest Import and Export Co., Ltd. China) were used as conductive wires. Finally, edible corn oil (Lion & Globe Corn Oil, Hop Hing Oils & Fats Company, Yuen Long, Hong Kong, China.) was used as liquid in the cell. It was observed that the filling liquid should not diffuse through the cell wall nor should it cause swelling of the PDMS. Corn (maize) oil was successfully used as a filling material within silicone elastomers [45,46], since the chemical component of corn oil makes it less affined to the silicone matrix. This small affinity or low solubility resulted from a major discrepancy in the cohesive energy density of the two materials [47]. Besides corn oil, silicone grease was also suggested for superior stability with SE [46]. However, it was predicted that the viscosity of silicone grease would cause much internal friction and energy dissipation, leading to significant mechanical hysteresis of the pressure sensor. In comparison, the shear modulus of liquid was zero, which implies least mechanical hysteresis.

**Figure 3:**
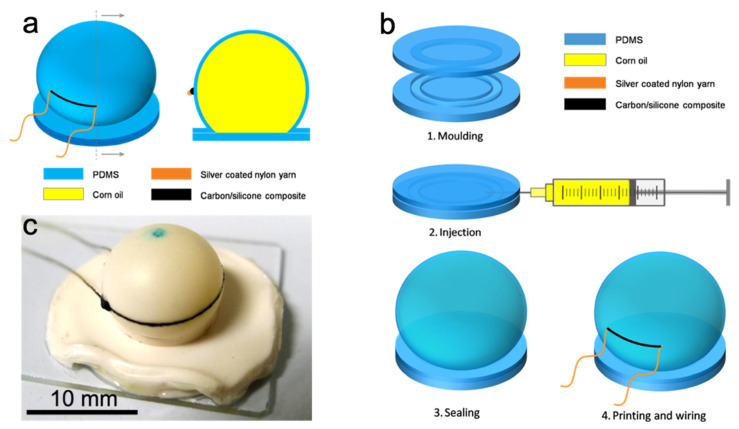
(**a**) Schematic of a cell-type pressure sensor. Left is an oblique view and right is a front view of the cross section. (**b**) Fabrication process of the cell-type pressure sensors. (1) Molding of the PDMS layers; (2) Injection of the corn oil; (3) Sealing of the cell; (4) Printing of the strain gauge and connection of the wires. (**c**) Picture of an as-made soft cell-type pressure sensor. The diameter of the cell is 11 mm. The green marking on top is for contraposition in fabrication.

A schematic diagram of the cell-type pressure sensor is shown in Figure 3a. The cell made from PDMS was filled with corn oil. The cell size can be adjusted by the volume of oil. On the cell wall, a carbon/silicone strain gauge was printed along the equator. Owing to the incompressibility of oil, when the cell was under compression, its equator was expected to expand greatly, leading to a stretch on the strain gauge. There were SCNYs connecting the strain gauge to external devices for resistance recording.

### 4.2. Fabrication

The carbon/silicone nanocomposite was prepared using a three-roll mill (PTR 65, Puhler, Köln, Germany) [37]. Then, a pair of PDMS layers was fabricated, as shown in Figure 3b. A sealing ring was deployed to ensure sealing of the conversion structure. The next step was to weigh 100 parts of SILASTIC^®^ 3483 Base and 7.5 parts of SILASTIC^®^ 83 Curing Agent in a clean container. After 2 min of intense mechanical mixing, the uniform mixture was cast onto two sets of PTFE molds and degassed in a vacuum chamber for 10 min in a 30 inHg vacuum. The material cured into two thick rubber layers within 24 h under ambient conditions. It may take another 6 days to reach final mechanical properties, during which the PDMS hardens. The final Young’s modulus was 230 kPa. The two layers excluding the sealing rings were 1 mm and 2 mm in thickness, respectively.

Next was the oil injection (Figure 3(b2)) and the sealing processes (Figure 3(b3)). After mold release, the bottom layer was pasted onto a clean glass slide using SE. By injecting corn oil between the two PDMS layers clamped together by splints with round perforations, a spherical cell was produced, that is, the conversion structure. Then, the whole structure was heated at 100 °C for 1 h to cure the SE (before injection, a small amount of SE was applied uniformly between the layers except the cell region), and glue the two layers together.

Finally, the conductive nanocomposite was printed onto the cell wall, and connected with wires (Figure 3(b4)). It is difficult to apply patterning technologies including ink-jet printing [48], screen printing [49], 3D printing [50], and photolithography (stencil printing) [51] here. Due to the high viscosity and poor fluidity of the nanocomposite, it is not viable for ink-jet printing and 3D printing. Neither stencil nor screen printing was applicable, because the conversion structure was spherical. Therefore, a method similar to offset printing was adopted. The next stage was to dip a SCNY into the composite, take the yarn out and keep it under a fixed tension, flip the yarn twice to remove excess mixture, and slowly move the strained yarn towards the spherical cell. When the yarn approached the cell, the paste was printed into a line on the equator. SCNYs were then embedded at two ends of the strain gauge and the composite was vulcanized at 100 °C for 5 min. An as-made soft pressure sensor on a glass slide is shown in Figure 3c.

The off-set printing determines the shape and size of the strain gauge, hence the sensing performance of the pressure sensor. To investigate the effect of printing, two types of SCNYs, 70D × 2 and 70D, were adopted. Among the samples made, we selected three representative ones, labelled samples A, B and C. Sample A was printed using 70D × 2 yarns, while B and C used 70D yarns.

## 5. Evaluation of Pressure Sensors

The three sensor samples were evaluated using the same coupled electromechanical setup as in Section 3. A load was applied where the specimen was compressed by 0.5–2.0 mm and released for five cycles. There were one-minute or ten-second intervals between the compression groups. A loading and unloading speed of 60 mm/min was used for all the cycles.

### 5.1. Sensitivity

**Figure 4:**
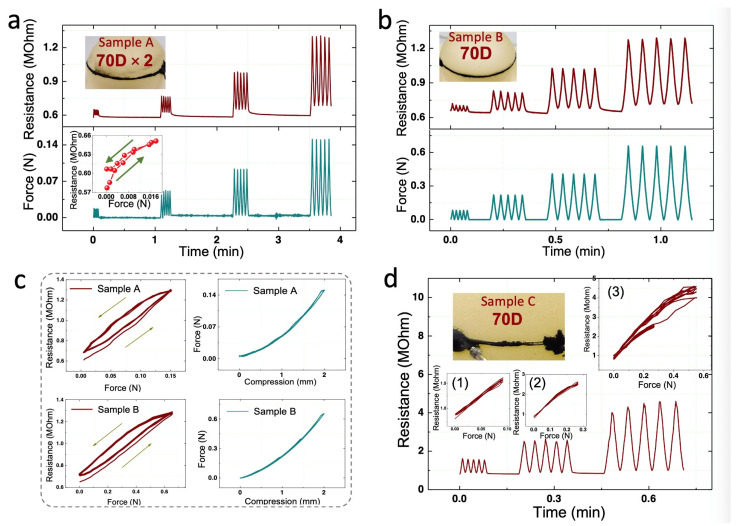
(**a**)Typical force and resistance curves of pressure sensor sample A under cyclic compression. The strain gauge, as pictured in the upper inset, was printed using 70D × 2 yarns. Lower inset is the force-resistance curve of the first 0.5 mm compressive cycle. (**b**) The force and resistance curves in cyclic compression tests. Inset is a picture of the sample B. Strain gauge on this sensor was printed using a 70D yarn. (**c**) Electrical and mechanical curves of A and B in the 2 mm compressions. (**d**) Resistance change of pressure sensor sample C. There were major defects on the gauge. (1) and (2) are force-resistance curves in the 0.5 mm and 1.0 mm cyclic compressions, respectively. (3) Force-resistance curves in 0.5 mm, 1.0 mm, and 1.5 mm cyclic compressions. The maximum force of 0.6 N corresponds to a pressure around 7 kPa.

Figure 4a shows typical force-resistance curves of soft pressure sensor, sample A. The PDMS layers were not fully hardened when being injected. The sample was highly sensitive to compression forces from 0 to 0.15 N (1.6 kPa) with the resistance doubled. The sensitivity was 0.7 kPa^−1^, higher than 0.55 kPa^−1^ of flexible pressure sensors with micro-structured rubber dielectric layers [10]. The lowest detectable force was 0.0012 N (13 Pa, see inset), which is triple the mass of a bee of 40 mg [52] and six times the mass of a bluebottle fly [10] or a feather of 20 mg [11]. The peak force in the first five compressive cycles was 0.018 N, corresponding to a pressure of 0.19 kPa (diameter of the cell structure was ~11 mm). Therefore, in terms of small force measurement, this type of sensor is approaching previously reported highly sensitive pressure sensors [10,11,52]. Most importantly, the sensors for benchmark are only flexible, neither soft nor stretchable; whereas our sensor is both soft and highly sensitive.

### 5.2. Pressure Measurement Range

The performance of a representative sensor printed using a 70D yarn is depicted in Figure 4b, where inset is a picture of sample B. The PDMS layers fully hardened when they were injected. Similar to sample A, this sensor was highly sensitive to forces from 0 to 0.65 N with the resistance doubled. The interval between compressive groups was set at 10 s so that the curves within each group can be shown more clearly in the panorama. It should be noted that the larger force measuring range of 0.65 N than that of sample A was not a result of the off-set printing process, but due to different rigidities of the cell structures. In the molding process, the mixture cured within 24 h. However, it takes another 6 days to reach final mechanical properties (according to product information from Dow Corning). The injection for sample A was carried out within 2 days of molding while sample B was 7 days after molding. Therefore, sample A had a lower rigidity. In this regard, the timing of injection can be adopted as a method to tune the pressure measuring range of the sensor, which may at least range between 0.15 N and 0.65 N (1.6–6.8 kPa). Besides, the measurement range can also be adjusted by changing the thickness of the upper layer and the volume of corn oil injected. An increase in either of the two parameters will lead to a larger rigidity of cell structure, hence a larger pressure range. For example, when only the former parameter is changed, a thicker upper layer will produce a thicker cell wall, and the overall hardness of the structure will increase, so as to withstand a larger normal pressure. Likewise, when more corn oil is injected, the cell will expand, stiffening the PDMS layer.

### 5.3. Mechanical Hysteresis

Although both samples, A and B, were highly sensitive to a low pressure, their time-dependent viscoelasticity incurred two adverse effects in electric output signals: relaxation behavior between compression cycles, and hysteresis behavior within compression cycles. As shown in Figure 4b, when the compressive load returned to zero at each cycle, the electrical resistance took a longer time to recover. In other words, the sensor had a noticeable electrical relaxation but a very small mechanical relaxation. Therefore, the electrical relaxation should come from the carbon/silicone strain gauge itself rather than from the oil-filled cell structure. Similarly, the electrical hysteresis values for the two samples were 16.5% and 18.6%, respectively, whereas mechanical hysteresis was too small to be neglected. Therefore, it was again confirmed that the electrical hysteresis arises from the carbon/silicone strain gauge rather than from the irreversible mechanical energy dissipation of the cell-type conversion structure.

### 5.4. Electromechanical Hysteresis

The resistive hysteresis of sample A was the lowest among those printed by 70D × 2 yarns, being around 30%. When scrutinizing the sample, we found that, different from the uniform printings of the others, there were variations of the width and thickness on the printed track. These variations were probably caused by excessive contact force during the printing process and the twist structure of the 70D × 2 yarns. In comparison, most sensors printed using 70D yarns showed a hysteresis between 15% and 20%, and the conductive printings were generally uniform in appearance. Based on the above results, it was hypothesized that a dimension effect may come into play: narrower and thinner printed tracks lead to a smaller hysteresis.

The hypothesis of dimension effect was tested and confirmed by sample C, the performance of which is summarized in Figure 4d. The sensor demonstrated a hysteresis as low as 7.7% in 0.5 mm and 1.0 mm cyclic compressions (see the two small insets). Almost all the resistance values came back along the same path, which was strikingly different from the pressure-resistance curves of the previous samples. According to the hypothesis, the strain gauge on this sensor should be sufficiently thin and narrow. As given in inset picture, there was a noticeable defect, an extremely thin and narrow part (~50 μm), on the right side of the strain gauge (printed using a 70D yarn). This major defect further verified the hypothesis of the dimension effect. It is also because of this defect that the initial resistance of sample C was around 1 MΩ, 1.67 times that of sample A and B, despite that the length of printing on C was only ~1/7 times those of the other two.

It is worth noting that the 7.7% hysteresis of sample C was very close to the resistance hysteresis of 8.08% for the carbon/silicone nanocomposite in Section 4, which suggests that this design of pressure sensor makes full advantage of this strain gauge. This low hysteresis of 7.7% has not only double confirmed the hypothesis of the dimension effect, but also validated the results in Section 4. In other words, the dimension effect is closely related with the amount of residual SO. Thinner printings lead to less residual SO, hence a low hysteresis.

### 5.5. Sample Consistency

Sample consistency is the uniformity among different sensor samples. In order to realize a pressure sensor array, as depicted in Figure 1c, it is important to ensure a high sample consistency. The sample consistency is a prerequisite to process signals from sensor arrays with normalized transfer functions. Unfortunately, in this study, the sensor samples showed a poor consistency in sensitivity. In this case, when sensors are used as a sensor array, each sensing element needs to be calibrated individually and a unique transfer function will be used for each element.

The low consistency mainly comes from the manual transfer printing process. The strain gauges printed in this method were inconsistent in length, width, thickness, and morphology, as demonstrated in Figure 4. Therefore, it is recommended to improve the uniformity of printing by exploring other printing techniques, such as stencil printing before cell formation and ink-jet printing after cell formation in future research.

## 6. Conclusions

In summary, we have designed, fabricated, and evaluated soft pressure sensors with a bioinspired conversion structure. The sensors with liquid-filled cells were highly sensitive to compression forces below 0.65 N (6.8 kPa). The lowest detectable force was ~0.0012 N (13 Pa), three times the mass of a bee [52] and six times the mass of a bluebottle fly [10] or a feather [11]. Sensitivity of the sensor can be as high as 0.7 kPa^−1^, even higher than 0.55 kPa^−1^ of flexible pressure sensors with micro-structured rubber dielectric layers [10]. Most importantly, the sensor was soft (elastic modulus below 230 kPa) rather than being only flexible. Pressure measurement range and sensitivity of the sensor can be easily tuned by changing the thickness of the cell wall, the volume of oil injected, and strain gauge parameters. Hysteresis of the sensors can be as low as 7.7% (or ±3.85%), owing to a very thin strain gauge, which also suggests the necessity of miniaturization towards micrometer scale. Besides, experiments in materials revealed that the electromechanical hysteresis of SE/SO/CB declined significantly from 38.5% to 8.08% after heat treatment, which agrees well with the lowest hysteresis of 7.7% for the pressure sensors.

Future work may include the application of microfluidic technology to manufacture smaller cell units and may focus on how to arrange strain-sensing elements on cells in a consistent and repeatable manner, as well as the packaging technology. Fully elastic connectors need to be developed to replace the silver plated yarns, so that the whole device can be both soft and stretchable as real skin.

## Figures and Tables

**Table 1:** Benchmark of the cell-structured sensor against typical sensors in the literature.

Soft/Flexible	Young’s Modulus (kPa)	Working Range (kPa)	Sensitivity (kPa^−1^)	Hysteresis	Research
Soft	30–70 *	0–10	—	0	Target
Soft	<230	0.013–6.8	0.7	7.7%	This work
Soft	2000	0.055–24	0.00087	6% **	[6]
Soft	63	25–50	0.25 **	28% **	[13]
Soft	—	0–124	0.59	—	[14]
Soft	—	0–30	—	—	[16]
Flexible	—	0–50	4.2	11.5%	[19]
Flexible	—	0–6	7.49	—	[21]
Soft	—	0–2	0.00952	~0%	[22]
Flexible	—	0–36	3.96	28.6%	[23]
Flexible	—	0–6.5	0.48	—	[24]
Soft	—	−60–20	0.0047	—	[25]
Soft	—	0–10	0.148	—	[26]
Soft	—	0.02–5	6.13	—	[27]

* Young’s modulus of the human skin [20]. ** Recalculated according to the pressure-resistance curves in the literature.

**Table 2:** Sample specification and processing parameters for strain gauge characterization.

Sample ID	Composition * (CB/SE/SO)	Dimension After Baking (mm)	Baking Time (hour)	Final Mass (mg)	Initial Resistance (kΩ)
#1–#5	0/100/0	0.48 (±0.02) × 50 × 20	0.42	500 (±16)	+∞
#6–#10	0/91/150	0.41 (±0.02) × 50 × 20	0.25	370 (±28)	+∞
#11–#15	0/91/150	0.36 (±0.03) × 42.6 (±0.19) × 17.3 (±0.19)	34	270 (±5)	+∞
#16–#20	9/91/150	0.45 (±0.01) × 50 × 20	0.25	490 (±9)	0.72 (±0.03)
#21–#25	9/91/150	0.45 (±0.04) × 43.5 (±0.4) × 17.6 (±0.2)	34	350 (±11)	1.71 (±0.09)

* The composition is displayed in weight ratio.

## Data Availability

Not applicable.
